# Intermittent restraint stress induces circadian misalignment in the mouse bladder, leading to nocturia

**DOI:** 10.1038/s41598-019-46517-w

**Published:** 2019-07-11

**Authors:** Tatsuya Ihara, Yuki Nakamura, Takahiko Mitsui, Sachiko Tsuchiya, Mie Kanda, Satoru Kira, Hiroshi Nakagomi, Norifumi Sawada, Manabu Kamiyama, Eiji Shigetomi, Youichi Shinozaki, Mitsuharu Yoshiyama, Atsuhito Nakao, Schuichi Koizumi, Masayuki Takeda

**Affiliations:** 1 Department of Urology, Interdisciplinary Graduate School of Medicine, University of Yamanashi, Chuo, Yamanashi, Japan; 2 Department of Immunology, Interdisciplinary Graduate School of Medicine, University of Yamanashi, Chuo, Yamanashi, Japan; 3 Department of Neuropharmacology, Interdisciplinary Graduate School of Medicine, University of Yamanashi, Chuo, Yamanashi, Japan

**Keywords:** Mechanisms of disease, Circadian rhythms, Physiology

## Abstract

Intermittent stress disrupts the circadian rhythm in clock genes such as *Per2* only in peripheral organs without any effect on the central circadian clock in the suprachiasmatic nucleus. Here, the effect of restraint stress (RS) on circadian bladder function was investigated based on urination behavior and gene expression rhythms. Furthermore, PF670462 (PF), a Per2 phosphorylation enzyme inhibitor, was administered to investigate the effects on circadian bladder re-alignment after RS. Two-hour RS during the light (sleep) phase was applied to mice (RS mice) for 5 days. The following parameters were then examined: urination behaviors; clock gene expression rhythms and urinary sensory-related molecules such as piezo type mechanosensitive ion channel component 1 (*Piezo1*), transient receptor potential cation channel subfamily V member 4 (*TRPV4*), and *Connexin26* (*Cx26*) in the bladder mucosa; Per2 expression in the excised bladder of *Per2*^*luciferase*^ knock-in mice (Per2::luc); *in vivo* Per2 expression rhythms in the bladder of Per2::luc mice. Control mice did not show altered urination behavior in the light phase, whereas RS mice exhibited a higher voiding frequency and lower bladder capacity. In the bladder mucosa, RS mice also showed abrogated or misaligned *Piezo1*, *TRPV4*, *Connexin26*, and clock gene expression. The rhythmic expression of Per2 was also altered in RS mice both in excised- and *in vivo* bladder, compared with control mice. After PF administration, voiding frequency was reduced and bladder capacity was increased during the light phase in RS mice; the *in vivo* Per2 expression rhythm was also fully restored. Therefore, RS can alter circadian gene expression in the bladder during the light phase and might cause nocturia via changes in circadian bladder function due the dysregulation of clock genes. Amending the circadian rhythm therapeutically could be applied for nocturia.

## Introduction

Clock genes such as *Per2*, *Bmal1*, and *Rev-erbα* as representatives regulate transcriptional and translational mechanisms in organisms with circadian rhythms. Circadian gene expression, generated by clock genes, regulates many aspects of behavior and physiological processes involving various metabolic enzymes, channels, and receptors, and abnormalities in clock genes have been reported to be associated with various diseases^1^. Lower urinary tract functions are also associated with the circadian rhythm and clock gene regulation. The sensation of bladder fullness has a circadian rhythm correlated with the gene expression of urinary sensory-related molecules such as piezo type mechanosensitive ion channel component 1 (*Piezo1*), transient receptor potential cation channel subfamily V member 4 (*TRPV4*), and *Connexin26* (*Cx26*) under the regulation of clock genes in the bladder mucosa, and lower urinary tract symptoms including nocturia are exacerbated with the dysregulation of urinary sensory-related molecules and clock gene abnormalities^2–7^.

People often encounter circadian misalignment associated with clock genes disorder (CMACD). These include not only living in modern society, which involves various stressors and inappropriate evening light-exposure, but also aging^8–12^. Further, shift workers are exposed to irregular sleep/wake cycles, which disturbs not only circadian rhythms involved in organ function but also clock genes at the epigenetic and transcriptional levels^13^. These CMACDs are linked to increased risks for physical and psychiatric disorders including cardiovascular disease, diabetes, obesity, cancer, and depression, among others^14–18^. Moreover, various factors have been reported to induce CMACD. For example, the administration of a high fat diet induces alterations to metabolic enzyme expression in the liver, which is accompanied by disruptions in the circadian expression of clock genes, resulting in disturbances in rhythmic liver metabolite concentrations^19^. The intestinal flora has been reported to show a circadian rhythm^20^. Accordingly, interruption of the circadian rhythm with respect to the bacterial flora can cause CMACD in the mouse liver^21^.

Furthermore, some types of intermittent stress such as restraint stress (RS) can disrupt the circadian rhythm of clock genes only in peripheral organs, without any effect on the central clock in the suprachiasmatic nucleus (SCN) in mice^22^. We hypothesized that RS could cause CMACD in the mouse bladder resulting in nocturia. In the present study, to reveal the effect of RS on alterations to circadian bladder function, we investigated its influence on urination behavior and gene expression rhythms in the mouse bladder.

## Results

### Restraint stress induces nocturia in mice

**Figure 1:**
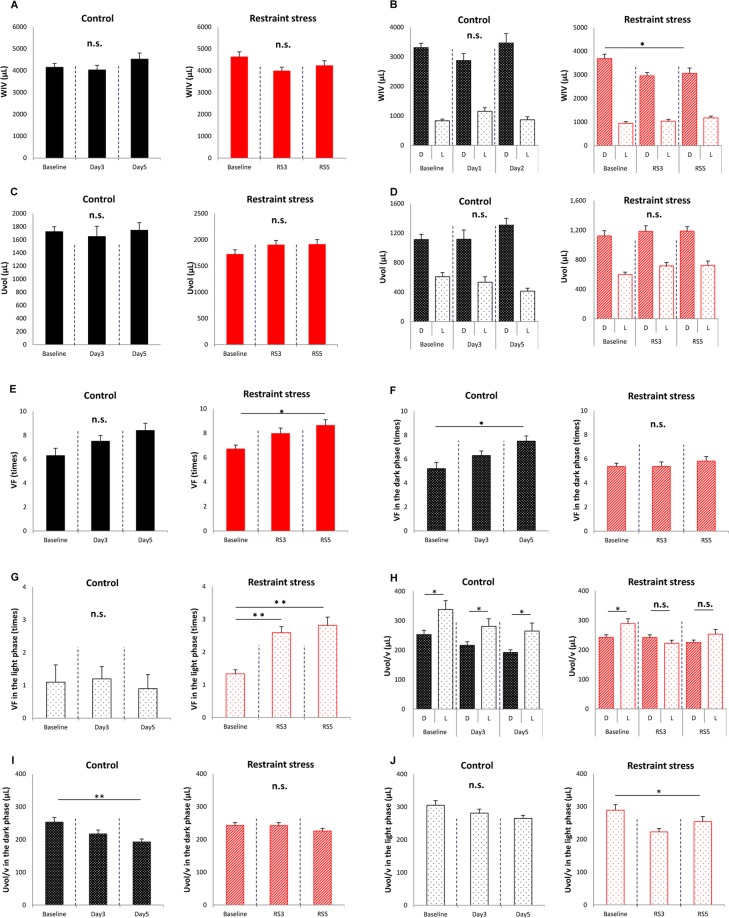
Differences in voiding behavior after restraint stress (RS). (**A**) Water intake volume (WIV) over 24 h in control and mice subjected to restraint stress (RS mice). (**B**) WIV in the dark (active) and light (sleep) phase in control and RS mice. (**C**) Total urine volume (Uvol) in control and RS mice. (**D**) Uvol in the dark and light phase in control and RS mice. (**E**) Total voiding frequency (VF) in control and RS mice. (**F**) VF in the dark phase in control and RS mice. (**G**) VF in the light phase in control and RS mice. (**H**) Urine volume/voiding (Uvol/v) in control and RS mice. (**I**) Uvol/v in the dark phase in control and RS mice. (**J**) Uvol/v in the light phase in control and RS mice. The horizontal axis represents the day of measurement (Supplementary Fig. 2). Data are presented as means ± standard error (SE). D; The dark phase. L; the light phase. Numbers of mice are 10 for control mice and 27 for RS mice. Statistical analyses were performed by a one-way ANOVA with Bonferroni’s test. The Mann-Whitney U-test was used to compare differences in Uvol/v between active and sleep phases. A *P* value less than 0.05 was considered significant. *P < 0.05, **P < 0.01, n.s., not significant.

Body weights were not different between control and mice subjected to restraint stress (RS mice) (25.57 ± 0.54 vs. 24.44 ± 0.36 g, respectively, P = 0.10, by student’s *t*-test, N = 10 and 27 for control and RS mice, respectively). Actogram showed that the locomotor activity pattern that mouse was active in the dark and rest/sleep in the light period was the same between control and RS mouse (Supplementary Fig. 1). Total water intake volume (WIV) was also not different between control and RS mice (Fig. 1A); however, when WIV in the dark (active) and light (sleep) phases were individually compared, control mice did not show a difference, whereas in RS mice, WIV in the dark phase for RS3, 3 days after RS loading (Supplementary Fig. 2), was significantly lower than baseline values (Fig. 1B). Total urine volume (Uvol) and Uvol in the dark and light phases were not different in control and RS mice (Fig. 1C and 1D).

Total voiding frequency (VF) was unchanged in control mice; however, in RS mice this was significantly higher on RS5 compared to baseline levels (Fig. 1E). VF in the dark phase gradually increased and that on Day 5 was significantly higher than baseline levels of control mice. However, RS mice did not show any differences in VF during the dark phase (Fig. 1F). In contrast, VF in the light phase was not different in control mice. However, that in the light phase increased significantly after RS, compared to baseline values (Fig. 1G).

Urine volume/voiding (Uvol/v) in the dark phase was significantly smaller than that in the light phase in control mice. In contrast, differences in Uvol/v between the dark and light phase that were observed at baseline disappeared in RS mice (Fig. 1H). In control mice, a tendency of decreased Uvol/v in the dark phase was also observed and values at Day 5 were significantly smaller than baseline levels. However, in RS mice, no differences were observed (Fig. 1I). In contrast, Uvol/v in the light phase was not different in control mice. However, that in the light phase was significantly lower on Day 3 in RS mice (Fig. 1J). The representative traces were shown in Supplementary Fig. 3.

### RS disrupts the gene expression rhythm in the mouse bladder mucosa

**Figure 2:**
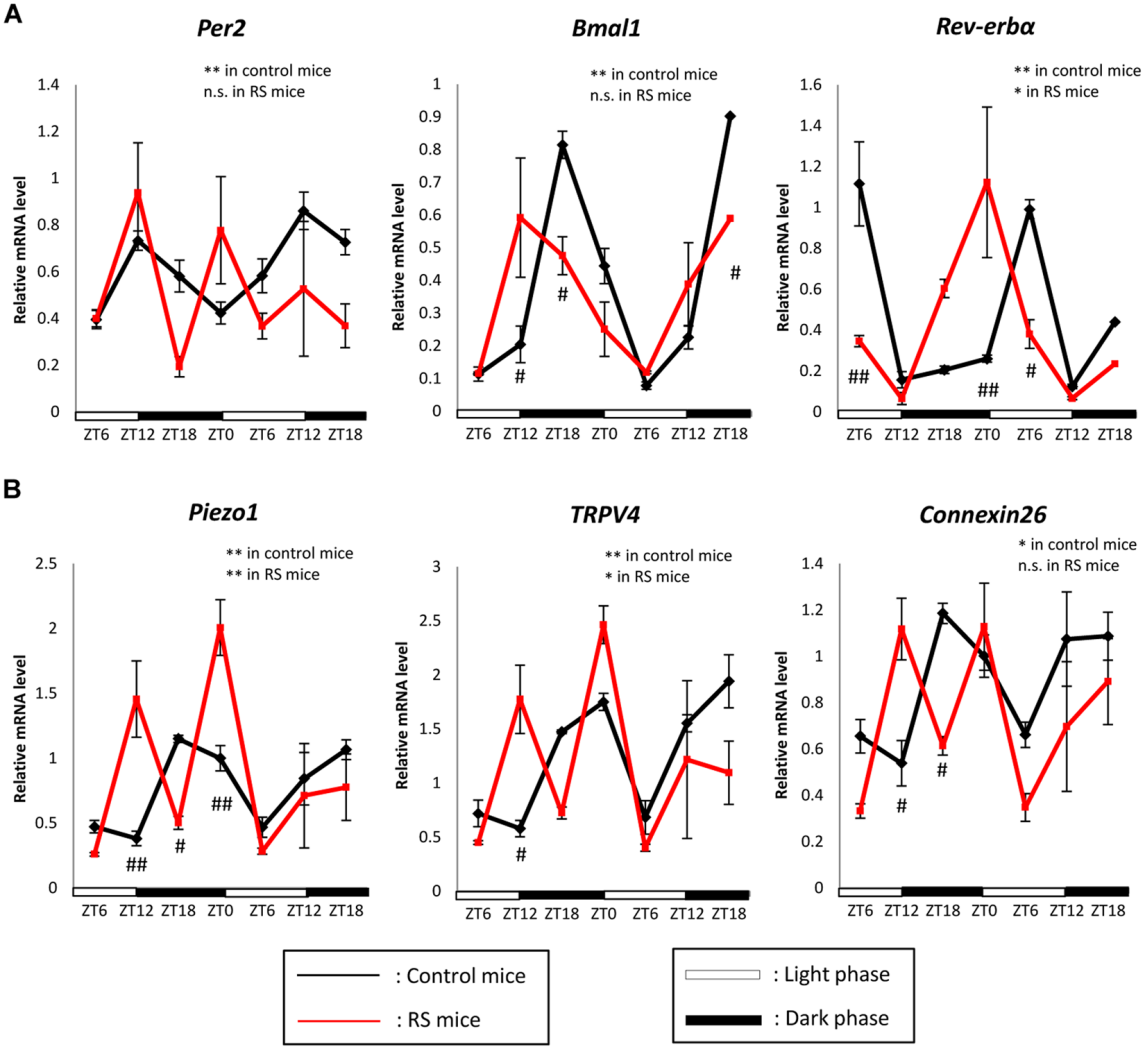
Gene expression rhythms in the mouse bladder mucosa. (**A**) Clock gene mRNA expression rhythms in the mouse bladder mucosa in control and mice subjected to restraint stress (RS mice). (**B**) Piezo type mechanosensitive ion channel component 1 (*Piezo1)*, transient receptor potential cation channel subfamily V member 4 (*TRPV4)*, and *Connexin26* (*Cx26)* mRNA expression rhythm in the mouse bladder mucosa in control and RS mice. The number of mice was 4 for both groups at each point. ZT; zeitgeber time. Statistical analyses were performed using a one-way ANOVA to compare differences among time points in each group. *P < 0.05, **P < 0.01, n.s., not significant. A two-way ANOVA with Bonferroni’s test was used to compare differences between control and RS mice at each time point. ^#^*P* < 0.05, ^##^*P* < 0.01. Data are presented as means ± SE.

In control mice, the expression in *Per2*, *Bmal1*, and *Rev-erbα* showed a typical circadian rhythm. In contrast, the circadian rhythms of Per2 and Bmal1 were disrupted in RS mice, Rev-erbα expression rhythm maintained time-dependent variation in RS mice. However, the peak expression time was shifted forward and circadian Rev-erbα expression was also disrupted in RS mice compared to that in control mice (Fig. 2A).

Urinary sensory-related molecules such as *Piezo1*, *TRPV4*, and *Cx26* also showed circadian gene expression in control mice. However, the expression rhythm of *Piezo1* and *TRPV4* showed a time-dependent change in RS mice and the expression pattern was different from that in control mice. Regarding *Cx26* expression, RS mice showed disrupted circadian rhythm (Fig. 2B).

### RS induces the circadian misalignment of Per2 expression in the mouse bladder

**Figure 3:**
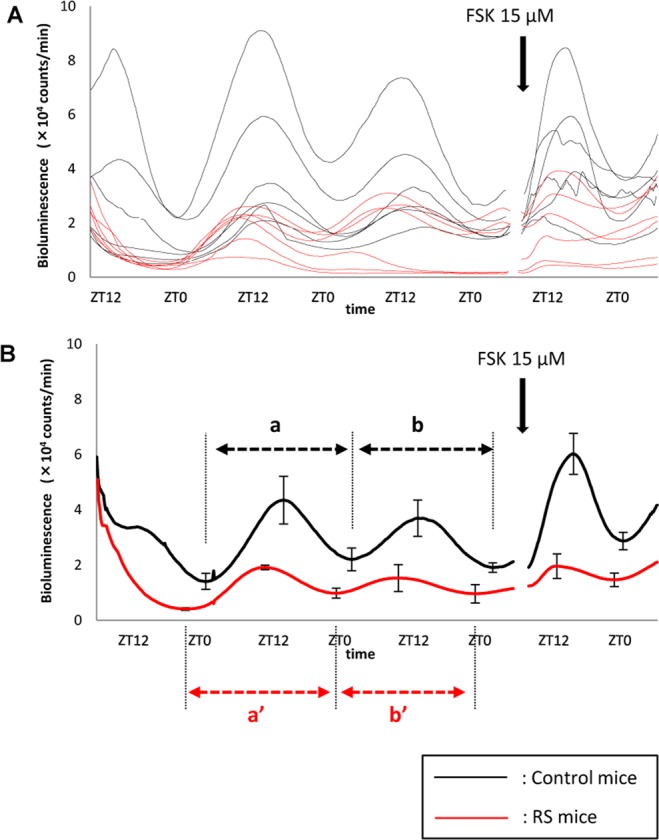
Per2 bioluminescence over time in the *ex vivo* mouse bladder. (**A**) Per2 expression rhythms in individual *ex vivo* mouse bladders for control and mice subjected to restraint stress (RS mice). (**B**) Mean values. The number of mice was 6 for both groups. ZT; zeitgeber time. Black arrow indicates the time of 15 μM forskolin administration, to confirm the viability of the excised bladder. Circadian period, a vs a’: 25.38 ± 0.30 vs 26.12 ± 0.33 h; P = 0.34. b vs b’: 24.35 ± 0.21 vs 25.12 ± 0.31 h; P = 0.29 based on the Mann-Whitney U-test. Amplitude in control and RS mice between the first nadir and peak: 30300 ± 6494 vs 15310 ± 2465 (photons/min), P = 0.045; between the second nadir and peak: 15661 ± 2695 vs 4876 ± 2011 (photons/min); P = 0.023 based on the Mann-Whitney U-test.

Before comparing the Per2 expression rhythm in the *ex vivo* bladder between control and RS mice, we investigated how excision of the bladder could affect the gene expression rhythm. When the bladder was excised at both zeitgeber time (ZT) 8 and ZT20, which are the peak and nadir time of *Per2* expression^3^, the expression rhythm was reset and a new circadian rhythm was established immediately after excision. The peak expression time in the bladders that were excised 12 h apart were similarly shifted by 12 h (Supplementary Fig. 4). Based on these findings, the Per2 expression rhythm was compared in the *ex vivo* bladder excised at ZT8 between control and RS mice. The circadian period was approximately 24 h in both groups. However, the amplitude of the circadian rhythm was significantly lower for RS mice (Fig. 3).

**Figure 4:**
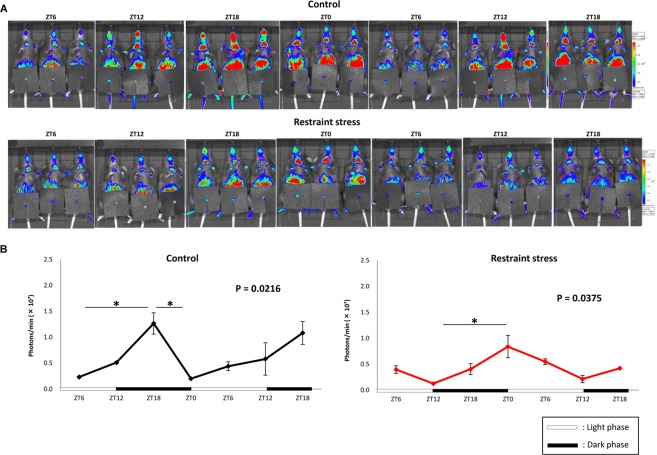
Per2 bioluminescence in the *in vivo* mouse bladder. (**A**) Images of Per2 expression in control and mice subject to restraint stress (RS mice). (**B**) The total region of interest (ROI) quantification of Per2::Luc bioluminescence in the bladder of control and RS mice. The amplitude from nadir to peak was 1.349 ± 0.002 × 10^7^ (photons/min) in control mice and 0.737 ± 0.219 × 10^7^ (photons/min) in RS mice; P = 0.049 based on the Mann-Whitney U-test. Three mice were used for each time point. ZT; zeitgeber time. Statistical analyses were performed using a one-way ANOVA with a Bonferroni’s test to compare differences among the time points in each group. A *P* value less than 0.05 was considered significant. *P < 0.05, n.s., not significant.

In *in vivo* imaging of mouse bladder, Per2-bioluminescence showed a circadian rhythm in both control and RS mouse bladders. However, the peak in Per2 expression, which was observed at ZT18 in control mice, was changed to ZT0 in RS mice. The amplitude of circadian rhythm was also lower in RS mice than in control mice (Fig. 4).

### PF670462 (PF) ameliorates circadian bladder dysfunction and nocturia induced by RS

**Figure 5:**
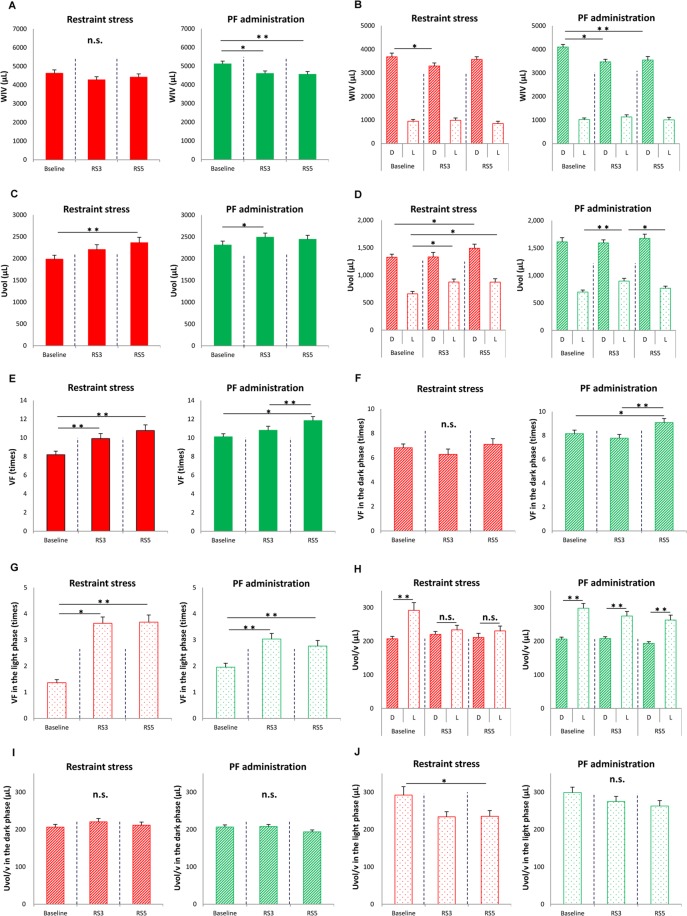
Voiding behavior after PF670462 (PF) administration. (**A**) Water intake volume (WIV) for 24 h in mice subjected to restraint stress (RS mice) and RS loading + PF administered mice (RS + PF mice). (**B**) WIV in the dark (active) and light (sleep) phases in RS and RS + PF mice. (**C**) Total urine volume (Uvol) in RS and RS + PF mice. (**D**) Uvol in the dark and light phase in RS and RS + PF mice. (**E**) Total voiding frequency (VF) in RS and RS + PF mice. (**F**) VF in the dark phase in RS and RS + PF mice. (**G**) VF in the light phase in RS and RS + PF mice. (**H**) Urine volume/voiding (Uvol/v) in RS and RS + PF mice. (**I**) Uvol/v in the dark phase in RS and RS + PF mice. (**J**) Uvol/v in the light phase in RS and RS + PF mice. The horizontal axis represents the day of measurement (Supplementary Fig. 1). Data are presented as the means ± standard error (SE). D; the dark phase. L; the light phase. Numbers of mice are 26 in RS mice and 24 in RS + PF mice. Statistical analyses were performed using a one-way ANOVA with a Bonferroni’s test. The Mann-Whitney U-test was used to compare the differences between two groups. A *P* value less than 0.05 was considered significant. *P < 0.05, **P < 0.01, n.s., not significant.

Next, the voiding behavior was compared between RS mice and RS mice administered 10 mg/kg PF (RS + PF mice). In this study, 100 μL deionized water (DW) was administered to RS mice intraorally for 5 days as a control. The body weight was the same between the two groups. (24.53 ± 0.57 vs. 25.41 ± 0.52 g, P = 0.16 based on a Student’s *t*-test; N = 26 and 24 for RS and RS + PF mice, respectively). Total WIV decreased significantly in RS + PF mice (Fig. 5A). However, both groups exhibited decreasing WIV in the dark phase and there were no changes in the light phase (Fig. 5B).

Total Uvol gradually increased and was significantly higher on RS5, as compared to baseline levels, in RS mice. In contrast, the increase in total Uvol was observed only on RS3 in RS + PF mice (Fig. 5C). Uvol in the dark and light phase was also significantly higher than baseline levels in RS mice. However, in the dark phase, Uvol did not show differences in RS + PF mice. Moreover, Uvol in the light phase was higher on RS3 than baseline levels in RS + PF mice, but then decreased significantly on RS5 (Fig. 5D).

Total VF increased significantly from baseline in both mice (Fig. 5E). VF in the dark phase was the same in RS mice. However, in the dark phase on RS5, VF was higher than baseline levels and that on RS3 in RS + PF mice (Fig. 5F). Further, VF in the light phase increased significantly from baseline in both groups. However, the increase in VF in the light phase from baseline was significantly lower in RS + PF mice than in RS mice on RS3 and RS5 (both P < 0.0001 between RS and RS + PF mice, based on Mann-Whitney U-test; Fig. 5G).

Uvol/v in the light phase was higher than that in the dark phase at baseline in both groups. Although this variation disappeared in RS mice on RS3 and RS5, RS + PF mice maintained higher Uvol/v in the light, compared to that in the dark phase at these time points (Fig. 5H). Uvol/v in the dark phase did not show any differences in both groups (Fig. 5I). However, Uvol/v in the light phase was lower on RS5 compared to baseline levels in RS mice and in contrast, RS + PF mice did not show differences in Uvol/v during the sleep phase (Fig. 5J). The representative traces were shown in Supplementary Fig. 3.

### Circadian misalignment in the mouse bladder after RS is ameliorated by PF

**Figure 6:**
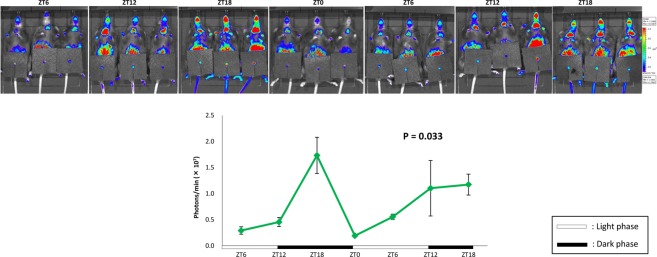
Effects of PF670462 (PF) on the circadian rhythm in the mouse bladder. The images and total region of interest (ROI) quantification of Per2::Luc bioluminescence based on the *in vivo* imaging of mouse bladders from the restraint stress loading + PF administered mice (RS + PF mice). Three mice were used for each time point. ZT; zeitgeber time. Statistical analyses were performed using a one-way ANOVA with a Bonferroni’s test to compare differences among the time points. A *P* value less than 0.05 was considered significant. n.s., not significant.

Based on the *in vivo* imaging of mouse bladders in the RS + PF group, Per2-bioluminescence showed a circadian rhythm (Fig. 6). Peak Per2 expression, which was observed at ZT18 in control mice and shifted to ZT0 in RS mice (Fig. 4), was restored to the timing observed in control mice in Fig. 4. Moreover, the amplitude of Per2 oscillation also seemed to restore to the control levels depicted in Fig. 4B.

## Discussion

The present study demonstrated that intermittent RS increases VF and reduces Uvol/v in the light (sleep) phase in mice. However, this treatment did not affect Uvol in the dark and light phase. Furthermore, RS mice exhibited alterations to the circadian rhythm of clock genes in the bladder, as well as *Piezo1*, *TRPV4*, and *Cx26* expression in the bladder mucosa, which could induce abnormal circadian bladder function and cause nocturia. Interestingly, these circadian misalignments in the RS mouse bladder were amended to control rhythms and nocturia was ameliorated after PF administration.

In organisms, stress-related information is properly integrated through neural networks in the central nervous system (CNS). Then, minimized stress responses are necessary to maintain homeostasis via stress effectors such as the hypothalamic-pituitary-adrenal axis and the sympathetic and parasympathetic arms^23^. The secretion of vasopressin from the pituitary gland, one of the factors that regulates Uvol in the kidney, increases after chronic stress^24^. In the present study, locomotor activity pattern (Supplementary Fig. 1) and Uvol did not change in control and RS mice (Fig. 1C and 1D). Furthermore, if water intake behavior was considered a rhythmic daily activity, no differences were observed in total WIV, as well as WIV in the dark and light phases, between control and RS mice; however, the RS group showed lower WIV in the dark only on RS3 (Fig. 1A and 1B). This suggests that 5 days of RS does not affect the central clock in SCN, as reported previously^22^. Differences in WIV and Uvol were observed between RS and RS + PF mice (Fig. 5A–D), unlike the results shown in in Fig. 1A–D. Accordingly, the administration of 100 µL of DW, administered to RS mice and used as a solvent for PF, could represent too large of a volume for mice; however, the differences in Uvol were larger in the light phase than in the dark phase (Fig. 5D). This is thought to be indicative of the fact that drinking during the sleep phase affects nocturia and/or nocturnal polyuria^25^.

The differences in VF and Uvol/v in control mice were more prominent in the dark phase than in the light phase. In contrast, RS mice showed the opposite results (Fig. 1F, 1G, 1I, and 1J). These results suggest that RS affects voiding behavior only in the light phase. We measured voiding behavior for 6 days in metabolic cages. Such a long period of housing could have been a stressor for mice and induces the differences in voiding behavior in the dark phase^26^. However, 12 h dark/light cycles, the pivotal circadian entrainment regulators, would create robust circadian rhythms in mice, which was represented by consecutive circadian locomotor activity (Supplementary Fig. 1) and WIV even in the RS^2,8^. Probably, the 12 h dark/light cycle would limit the effect of the stress to the light phase. Therefore, RS mice showed nocturia pattern and did not show the voiding differences in the dark phase. RS might be more stimulating than housing stress. WIV and locomotor activity pattern were the same as control mice, result in offsetting the changes of voiding in the dark phase. However, control mice showed higher VF and lower Uvol/v only in the dark phase because of the housing stress during they are active, although the circadian rhythm created by the appropriate environment could leave urinations as regular pattern during they are rest/sleep.

Acute and temporal physiological stresses have been reported to activate the immune system. These stress signals can exert an anti-inflammatory effect and protect organs against ischemia by elevating plasma corticosterone levels^27^. However, 5 days of RS induced CMACD, as reported previously^22^. Even with the first RS, which corresponded to the acute stress phase, changes in voiding behavior such as higher VF and lower Uvol/v were observed (Fig. 1E and 1J). These results suggest that the bladder has lower stress tolerance than the kidney.

Uvol/v was previously reported to exhibit a circadian rhythm under the regulation of clock genes in the mouse bladder, which was enhanced in the sleep phase compared to that in the active phase^2,7^. The disruption of bladder capacity differences between day and night is speculated to be one cause of nocturia that is associated with circadian clock disorders in humans^28–30^. These changes were also observed in the present study (Figs 1H and 5H). Furthermore, we proved that RS is accompanied by CMACD in the bladder (Figs 2–4), whereas in PF-administered mice, the disrupted rhythm in Uvol/v and clock genes was restored (Figs 5H and 6). These results strengthen the relationship between nocturia and abnormalities in clock genes.

Urinary sensory-related molecules such as *Piezo1*, *TRPV4*, and *Cx26*, which create a circadian rhythm underlying the sensation of bladder fullness under the regulation of clock genes^3,5,6^, as well as clock genes, showed a circadian rhythm in the bladder mucosa of control mice. However, these rhythms were disrupted in RS mice (Fig. 2). These results indicate that RS mice suffer from CMACD in the bladder mucosa, underlying the sensation of bladder fullness, which might be one of the causes of nocturia in these mice^25^.

We also measured Per2 expression rhythms in both the *ex vivo* and *in vivo* bladder. The *ex vivo* bladder sustained circadian Per2 expression for several days as reported previously^31^, and a circadian rhythm was observed in control and RS mice. In addition, the amplitude in Per2 oscillation was significantly lower in RS mice (Figs 3 and 4). These results indicate that the circadian clock in the bladder was not completely disrupted, but rather partially damaged, by RS. In situations in which the regular clock function is completed disturbed, the circadian expression of clock genes should disappear. Furthermore, the function of clock genes remained lower in the bladder after the release from RS (Figs 3 and 4). It was suggested that changes in the circadian bladder function were not evoked temporally and immediately by stress-associated factors^23^; however, RS, for which effects accumulated gradually, is thought to affect the gene expression rhythm in the bladder by regulating clock genes.

Various stressors are suggested to affect urination in mice. Previously, it was shown that water avoidance stress leads to the development of pollakisuria, which is accompanied by the activation of immune cells in the bladder^32^. With social stress in mice, increased voiding was found to be accompanied not only by changes in local receptors in the bladder, but also by afferent nerve activity^33^. Moreover, physiological stress can cause an overactive bladder in humans^34^. Psychiatric stress, for which the causes are ambiguous, such as anxiety and post-traumatic-stress-disorder, can also increase nocturnal voiding^35,36^. These reports suggested that an unknown factor involving stress affects urination. In addition to stress-activated bladder function, CNS sensitivity, temperature, and irregular lifestyles can promote urination^37,38^. Interestingly, the circadian clocks in local organs are affected by these factors, often leading to various symptoms^1,39^. Therefore, RS-related voiding changes might be affected by various factors. We only suggest a relationship between stress and the circadian clock in the bladder as a possible cause of nocturia and have not investigated the detailed molecular changes occurring in response to RS that cause dyssynchronous clock gene expression, which exist in almost all cells; moreover, the precise circadian clock is not only determined by links between the CNS and peripheral nerves but also cell-to-cell circadian regulatory mechanisms^7,40,41^. Jet lag syndrome is caused by CMACD in the CNS, and results in an oscillatory shift in the circadian rhythm^42^. Therefore, RS was considered to induce CMACD in the bladder not only through the production of stress-associated hormones but also through neural network pathways. Further investigations are needed to reveal the mechanism underlying the relationship between the circadian clock and nocturia.

In the present study, stress-affected bladder function was investigated using RS, which was applied at the same time point, namely the light phase, in mice, forcing the animals to wake up. This might affect the evaluation of nocturia. Although stress in the dark phase does not reportedly exaggerate CMACD^22^, RS in the dark phase might have different effects than that in the light phase, unrelated to circadian function in the bladder.

Higher-dose PF administration was reported to cause a loss in clock gene function in mice^43^. In addition, 100 mg/kg acute PF administration was reported to increase the mortality rate in these animals^44^. However, the intraperitoneal injection of 50 mg/kg PF, albeit only once, can successfully suppress allergic reactions in mice, and this was found to be under the regulation of clock genes, preventing effects on the SCN such as melatonin secretion and dark and light perception^44,45^. Local PF administration via inhalation was also reported to be effective without the development of any adverse effects^46^. Based on our results, intraoral PF administration resulted in improved nocturia even at lower doses, specifically 10 mg/kg. Although it was only partial, the effect of PF, reduced VF and increased Uvol/v in the light, was observed from day 3 of administration (Fig. 5G, 5H and 5J). It seems that setting the appropriate administration time according to changes in the circadian rhythm might have maximized the effects of the smaller dose, resulting in a potential circadian-modulating therapy for the treatment for nocturia, which is currently refractory^47^.

In conclusion, we indicated that RS affected circadian regulation system between the central clock in SCN and peripheral clocks, which induced CMACD in the mouse bladder and could be associated with nocturia. Thus, in modern society, coping with stress and other CMACDs might reduce nocturia. We believe that the present study suggests a novel aspect of nocturia and could lead to the development and further understanding of a new strategy for the treatment of nocturia.

## Materials and Methods

### Animals

Eight-to-twelve-week-old male C57BL/6 mice and age- and sex-matched C57BL/6 *Pe2*^*Luciferase*^ knock in (Per2::Luc) mice were used for the following experiments. Mice were housed under 12-h light/dark conditions for 2 weeks before experiments with free access to food and water. All procedures were conducted in accordance with the Guiding Principles in the Care and Use of Animals in the Field of the Physiologic Society of Japan and the policies of the Institutional Animal Care and Use Committee. In addition, all experimental protocols were approved by the Animal Care Committee of the University of Yamanashi (Chuo, Yamanashi, Japan).

### Application of restraint stress

Mice were subjected to RS for 2 h from ZT4 to ZT6, corresponding to the sleep phase and the time period at which circadian misalignment was most likely^22^; this was performed by enclosing the animals in a metal mesh of 12 × 12 cm (Supplementary Fig. 5). RS was applied for 5 days (from RS1 to RS5; Supplementary Fig. 2).

### Metabolic cages

Voiding behaviors were compared between control and RS mice using metabolic cages. The precise urine collection and the artifacts elimination due to the drop of feces and morsels of food are capable by the use of mesh in the flooring of the cage^2,48^. The following parameters were evaluated: WIV (µL), Uvol (µL/24 h), Uvol/v (µL), and VF (number of times). After mice were acclimatized for 2 days in the cage, these parameters, including baseline levels, were recorded for 6 days (Supplementary Fig. 2) and compared between baseline, third day, and fifth day. Undisturbed voiding behavior was measured for 6 days using control mice. Urination during the light (sleep phase) was considered nocturia in mice. The definition of nocturia followed a previous report^2^.

### PF670462 (PF) administration

A selective casein kinase Iε/δ inhibitor, PF670462 (PF; Tocris Bioscience, Ellisville, MO), was diluted with 100 μL of DW and administered intraorally (10 mg/kg) at the same time of RS loading after aesthesia using sevoflurane. This compound is known as a Per2 phosphorylation enzyme inhibitor that leads to the retention of nuclear Per2 and delays in the expression of clock genes comprising the circadian cycle^43–45^. Further, 100 μL of DW was administered to RS mice for 5 days as a control.

### Quantitative real-time reverse transcription polymerase chain reaction (qRT-PCR)

RNA was extracted from mouse bladder mucosa every 6 h from ZT0 in control and RS mice and qRT-PCR assays were performed using the same primer sequences, reagents, and protocol as reported previously^3,4^. Representative clock genes including *Per2, Bmal1, Rev-erbα*, and urinary sensory-related molecules such as *Piezo1*, *TRPV4*, and *Cx26* were measured. The mRNA level was calculated from the standard curve, which was run simultaneously with the sample tubes, and was normalized to *Eif2a/Tbcc* concentrations^49^.

### *Ex vivo* measurement of Per2 bioluminescence in the bladder

Male C57BL/6 Per2::Luc mice were used for this. The bladder was excised from these animals at ZT10, which is the peak time of *Per2* expression^3^. The excised bladder was put in a 3.5-cm dish (Thermo Fisher Scientific, Waltham, CA) with phenol red-free Dulbecco’s Modified Eagle Medium (DMEM; WAKO, Tokyo, Japan) with 1% penicillin/streptomycin (P/S; Gibco^®^, Waltham, CA), 1% amphotericin B solution (AMPB, Sigma-Aldrich, St Louis, MO), and 0.2 μM Beetle Luciferin Potassium Salt (Promega, Madison, WI). Then, samples were immediately placed into a dish type luminometer (Kronos DioAB-2550; ATTO, Tokyo, Japan), and incubated under humidified conditions with 5% CO_2_ at 37 °C. The bioluminescence from excised bladder tissues was measured for 3 days at 10-min intervals. The Per2 expression rhythm was then compared between control and RS mice. To confirm the viability of bladder tissue, 15 μM of forskolin (Sigma-Aldrich) was added to the dish for 2 h and the medium was replaced, and measurements were restarted.

### *In vivo* imaging of bladder-Per2 bioluminescence

The bladders from Per2::Luc mice were exposed, and the urine was discharged by puncturing the top of the bladder with a 27-G needle under anesthesia with isoflurane. A black plastic plate was then inserted between the skin and the exposed bladder to mask background bioluminescence (Supplementary Fig. 6). The mice were laid on their back, and 5 mg/kg of Beetle Luciferin Potassium Salt (Promega) was injected subcutaneously into the back near the neck; 5 min later, Per2-bioluminescence from the exposed bladder, which was marked with a region of interest (ROI), was measured every 6 h for 60 s from ZT6 using an *in vivo* imaging system (Perkin Elmer, Waltham, MA) for control, RS, and RS + PF670462-administered mice.

### Statistical analyses

The experimental values were expressed as the means ± standard error (SE). The significance of differences between two groups was analyzed using the Mann-Whitney U-test. A one-way ANOVA with a Bonferroni’s test was used to compare differences among time points in each group. A two-way ANOVA with a Bonferroni’s test was used to compare differences at each time point between two groups. A *P* value less than 0.05 was considered significant.

## Supplementary information

Dateset 1 - 6
