# Author Correction: Intermittent restraint stress induces circadian misalignment in the mouse bladder, leading to nocturia

**DOI:** 10.1038/s41598-019-53132-2

**Published:** 2019-11-08

**Authors:** Tatsuya Ihara, Yuki Nakamura, Takahiko Mitsui, Sachiko Tsuchiya, Mie Kanda, Satoru Kira, Hiroshi Nakagomi, Norifumi Sawada, Manabu Kamiyama, Eiji Shigetomi, Youichi Shinozaki, Mitsuharu Yoshiyama, Atsuhito Nakao, Schuichi Koizumi, Masayuki Takeda

**Affiliations:** 1 Department of Urology, Interdisciplinary Graduate School of Medicine, University of Yamanashi, Chuo, Yamanashi, Japan; 2 Department of Immunology, Interdisciplinary Graduate School of Medicine, University of Yamanashi, Chuo, Yamanashi, Japan; 3 Department of Neuropharmacology, Interdisciplinary Graduate School of Medicine, University of Yamanashi, Chuo, Yamanashi, Japan

Correction to: *Scientific Reports* 10.1038/s41598-019-46517-w, published online 11 July 2019

This Article contains errors in Figure 4, where the incorrect image was used for panel 6 of Figure 4A (control at ZT12), which also affected Figure 4B. The correct Figure 4 appears below.

**Figure 4:**
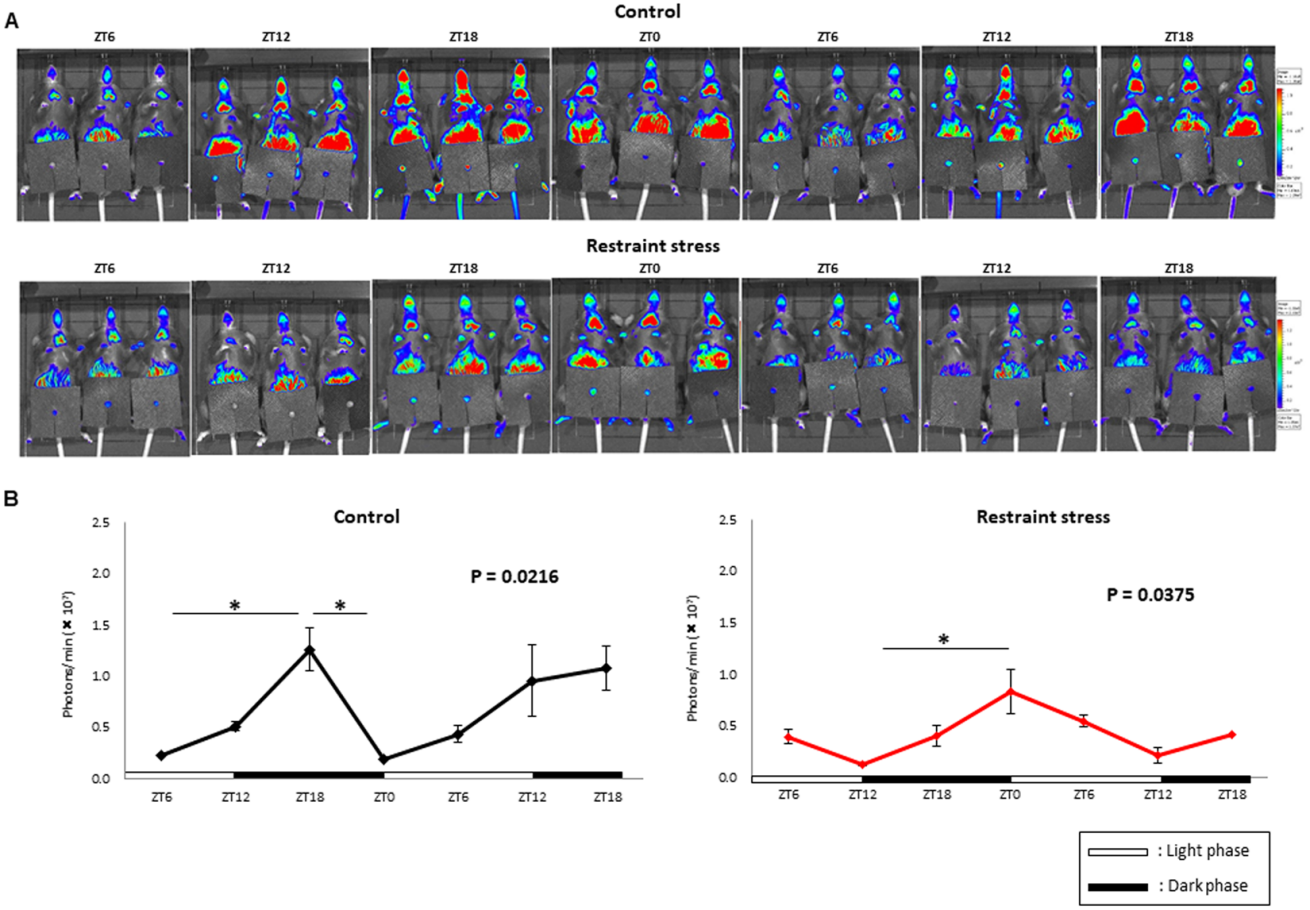
.

The conclusions of the Article are unaffected by these changes.

