# Supplementary material for: Intermittent restraint stress induces circadian misalignment in the mouse bladder, leading to nocturia
Source: Sci Rep. 2019 Jul 11;9:10069. doi: 10.1038/s41598-019-46517-w (PMC6624370; doi:10.1038/s41598-019-46517-w)
Supplement: Supplementary file 1 — Dateset 1 - 6 [file 41598_2019_46517_MOESM1_ESM.docx]

**Intermittent restraint stress induces circadian misalignment in the mouse bladder, leading to nocturia**

Tatsuya Ihara^1^, Yuki Nakamura^2^, Takahiko Mitsui^1*^, Sachiko Tsuchiya^1^, Mie Kanda^1^, Satoru Kira^1^, Hiroshi Nakagomi^1^, Norifumi Sawada^1^, Manabu Kamiyama^1^, Eiji Shigetomi^3^, Youichi Shinozaki^3^, Mitsuharu Yoshiyama^1^, Atsuhito Nakao^2^, Schuichi Koizumi^3^ and Masayuki Takeda^1*^

^1^Department of Urology, Interdisciplinary Graduate School of Medicine, University of Yamanashi, Chuo, Yamanashi, Japan

^2^Department of Immunology, Interdisciplinary Graduate School of Medicine, University of Yamanashi, Chuo, Yamanashi, Japan

^3^Department of Neuropharmacology, Interdisciplinary Graduate School of Medicine, University of Yamanashi, Chuo, Yamanashi, Japan

**
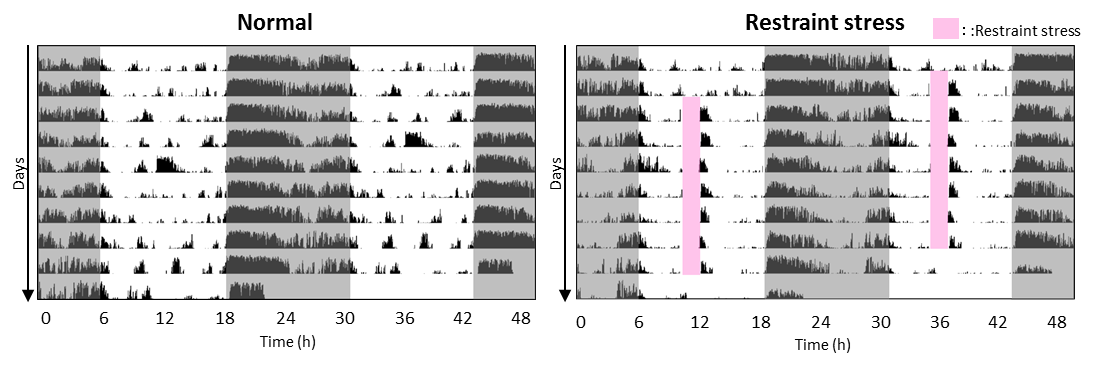
**

**Supplementary Figure 1**

Locomotor activity in control and restraint stress (RS) mice. Mice in cages were placed into an isolation rack to which an infrared sensor was attached on the celling (T-CH-M-LE, TOKIWA, Tokyo, Japan) under the 12hr dark/light period with free access to food and water. Mouse movement was detected by the infrared sensor, which were accumulated for 10 min, and recorded for 10 days, then calculated with actograms using ClockLab (Actimetrics, Wilmette, IL). RS was applied from Zeitgeber time (ZT) 4 to ZT6 for 7 days.

**
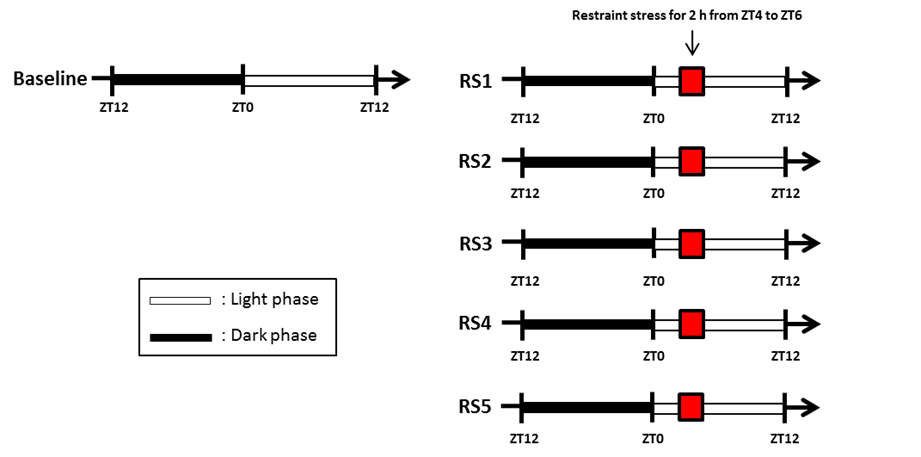
**

**Supplementary Figure 2**

The protocol of restraint stress. Restraint stress (RS) was applied from Zeitgeber time (ZT) 4 to ZT6 for 5 days (from RS1 to RS5). The voiding behavior was measured for 6 days continuously (through baseline to RS5).


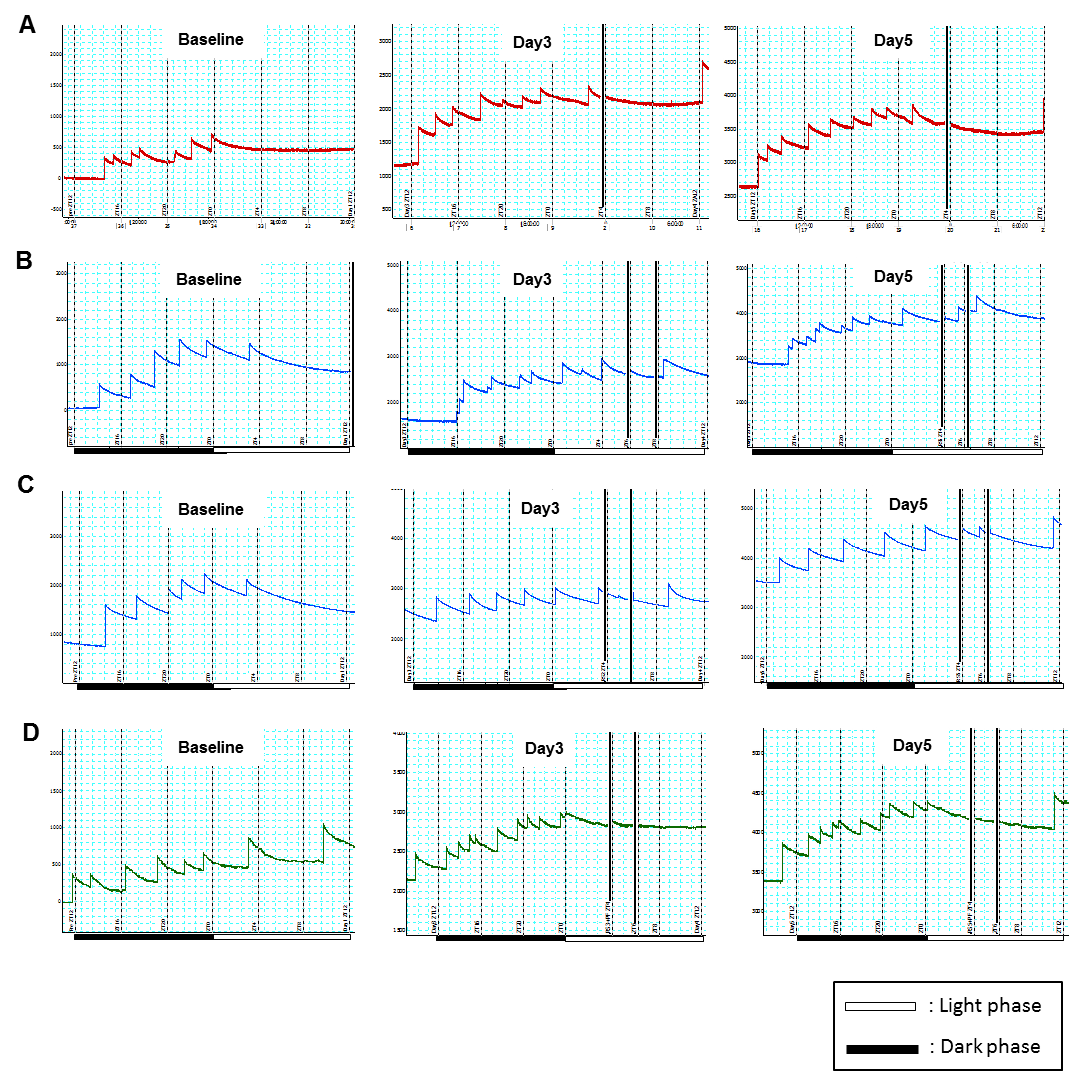


**Supplementary Figure 3**

The representative traces of mice voiding at baseline, third day, and fifth day. (A) Undisturbed voiding behavior (control mouse). (B) Restraint stress (RS) mouse. (C) RS mouse (vehicle of PF treated mice). (D) RS mice administered 10 mg/kg PF.


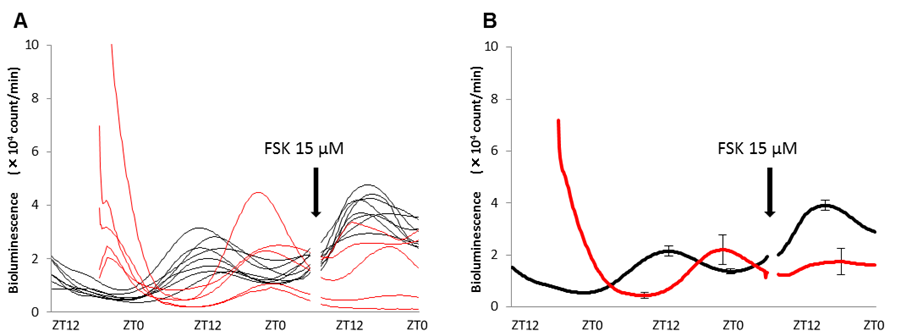


**Supplementary Figure 4**

Effect of bladder excision on gene expression rhythm. (A) Per2 expression rhythm in individual ex vivo mouse bladders. (B) Mean values. The bladder was excised at Zeitgeber time (ZT) 8 (the black line) and at ZT20 (the red line). The numbers of mice were 8 and 5 for bladders excised at ZT8 and ZT20, respectively. The black arrow indicates 15 μM forskolin administration.


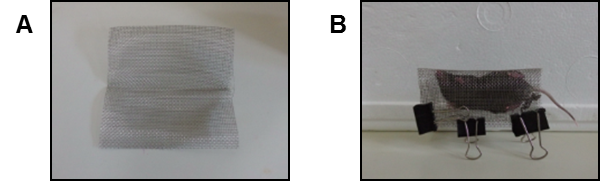


**Supplementary Figure 5**

Example of restraint stress. (A) A Metal mesh of 12 × 12 cm was used. (B) Image of the enclosed mouse. Urination behavior was continuously measured under this condition.


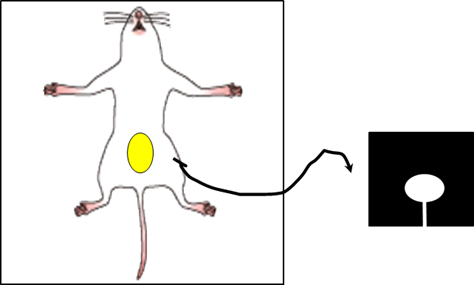


**Supplementary Figure 6**

Mouse bladder setting for in vivo imaging. A black plastic plate of 3.5 × 3.5 cm was inserted between the abdomen and the exposed bladder.
